# Role of Dietary Antioxidants in the Preservation of Vascular Function and the Modulation of Health and Disease

**DOI:** 10.3389/fcvm.2017.00064

**Published:** 2017-11-01

**Authors:** Saradhadevi Varadharaj, Owen J. Kelly, Rami N. Khayat, Purnima S. Kumar, Naseer Ahmed, Jay L. Zweier

**Affiliations:** ^1^Abbott Nutrition, Columbus, OH, United States; ^2^Division of Cardiovascular Medicine, Department of Internal Medicine, College of Medicine, Davis Heart & Lung Research Institute, The Ohio State University, Columbus, OH, United States; ^3^The Sleep Heart Program, Division of Pulmonary Critical Care and Sleep, Columbus, OH, United States; ^4^College of Dentistry, The Ohio State University, Columbus, OH, United States; ^5^Abbott Laboratories, Chicago, IL, United States

**Keywords:** antioxidants, endothelial nitric oxide synthase coupling, nitric oxide, blood flow, vascular health

## Abstract

In vascular diseases, including hypertension and atherosclerosis, vascular endothelial dysfunction (VED) occurs secondary to altered function of endothelial nitric oxide synthase (eNOS). A novel redox regulated pathway was identified through which eNOS is uncoupled due to *S*-glutathionylation of critical cysteine residues, resulting in superoxide free radical formation instead of the vasodilator molecule, nitric oxide. In addition, the redox sensitive cofactor tetrahydrobiopterin, BH_4_, is also essential for eNOS coupling. Antioxidants, either individually or combined, can modulate eNOS uncoupling by scavenging free radicals or impairing specific radical generating pathways, thus preventing oxidative stress and ameliorating VED. Epidemiological evidence and dietary guidelines suggest that diets high in antioxidants, or antioxidant supplementation, could preserve vascular health and prevent cardiovascular diseases (CVDs). Therefore, the purpose of this review is to highlight the possible role of dietary antioxidants in regulating eNOS function and uncoupling which is critical for maintenance of vascular health with normal blood flow/circulation and prevention of VED. We hypothesize that a conditioned dietary approach with suitable antioxidants may limit systemic oxidation, maintain a beneficial ratio of reduced to oxidized glutathione, and other redox markers, and minimize eNOS uncoupling serving to prevent CVD and possibly other chronic diseases.

## Introduction

Atherosclerosis and cardiovascular disease (CVD) have a large impact on society. Nearly half of the population of the United States has at least one of the major risk factors for heart disease which includes high blood pressure, elevated low density lipoprotein (LDL) cholesterol, aging and smoking (1), as well as possibly other modifiable risk factors such as type 2 diabetes, obesity, diet, lack of physical activity, and vascular endothelial dysfunction (VED) (2, 3). Drug therapies such as cholesterol lowering or antihypertensive drugs target only a given risk factor, so multiple drugs are required as more risk factors are present. However, various nutrients and antioxidants can primarily protect against VED (4, 5) and can address multiple risk factors, suggesting that a comprehensive nutritional regimen could be designed to target many of the risk factors that contribute to VED and CVD.

The excellent early review by Vita (5), and other reviews support a protective role of fruit polyphenols (6). In addition, the FLAVIOLA Health Study (FHS) showed that cocoa flavanol supplementation for 1 month (450 mg) improved the Framingham Risk Score in relatively healthy men and women (7), and a follow up study showed an improvement in endothelial function (as measured by flow-mediated vasodilation) in younger and older males (8). Another interesting study showed that geographic location and health status may influence outcomes of flavanol supplementation, in a meta-analysis of 18 human trials, although lipid profiles and CVD risk improved (9). This latter study suggests that other dietary factors (and possibly lifestyle) may be important as geographic location may greatly affect dietary intake. This is supported by a review showing dietary patterns, individual nutrients, and other dietary components can benefit endothelial function (10). There are many other recent reviews available covering-specific compounds, fruits, and other foods such as chocolate; however, discussing these is beyond the scope of this hypothesis paper. There is evidence that improving dietary intake of vegetables, nuts, and shifting to a Mediterranean-type diet (away from the Western diet), while reducing trans fatty acids and high glycemic foods can decrease the risk of CVD (11). It has been further suggested that poor dietary patterns or excess consumption of certain foods may be a risk factor for CVD (12).

While questions remain regarding the metabolic alterations that lead to CVD, oxidative stress/redox-imbalance has been implicated as a central mechanism of CVD (13). There is an abundance of literature, both positive and negative, available focusing on the effects of numerous plant/food-derived antioxidants on CVD. The purpose of this review is not to summarize this extensive and detailed literature on trials of antioxidants in CVD, as this has already appeared in prior reviews (14, 15). However, the aim is to put forth and examine the proposal that a novel central mechanism may exist by which dietary antioxidant compounds could reduce the risk of CVD.

It is clear that antioxidants serve to quench superoxide and secondary oxidants, maintain a high glutathione (GSH) to oxidized glutathione (GSSG) ratio (GSH:GSSG ratio) and, at the cellular level, this redox modulation could be beneficial in preventing VED that contributes to the onset of CVD (16–21). The conclusion that there is no benefit for antioxidants in CVD may be premature in that physiologically humans utilize numerous antioxidant compounds in many metabolic pathways. It may be that supplementing one antioxidant compound, or several known antioxidant compounds, for a short period (relative to the human lifespan) helps physiologically but may not be reflected in clinically measured endpoints. Furthermore, diet quality throughout the trials are rarely (if at all) assessed, suggesting some other dietary factors may be obscuring the effect of a given antioxidant supplement. Moreover, CVD is a chronic disease and takes years or decades to develop. In addition, it is unknown what specific initiating metabolic changes lead to CVD. It may simply be that the factors and oxidative stress promoting CVD events outweigh the antioxidant effect expected from the addition of one or more compounds. In clinical trials, it must also be expected that nutrients and other compounds in foods are multifunctional and do not target one pathway; as would be the case in a pharmaceutical trial. Many questions remain and therefore, well-designed nutritional studies will be required considering the novel molecular targets.

The most widely known nutritional guidelines for the prevention of CVD are published by the American Heart Association/American College of Cardiology (AHA/ACC). The most recent (2013) dietary guidelines include the following: diet high in fruits, vegetables and whole grains, nuts and legumes, and non-tropical vegetable oils. This dietary advice, within appropriate caloric intakes, is based on the Mediterranean (MED), Dietary Approach to Stop Hypertension (DASH), AHA, and United States Department of Agriculture (USDA) dietary plans (22). It has been reported that nutrition could improve blood flow/vascular tone through improvements in endothelial function or control of blood pressure as evidenced by studies in which diets high in fruits and vegetables have been associated with reduced risk of CVD and mortality (23, 24). In addition to this, there is the concern that the overall intake of fruits and vegetables are low in the United States, resulting in a decrease in the intake of antioxidants (25). These data showed that the mean intakes of vitamin E and selenium are below the Dietary Reference Intakes, while no recommended intakes exist for carotenes and flavonoids. Therefore, the optimal intake levels of antioxidants are unknown and while this is thought to impact on the risk of CVD, the best diet and dietary supplements to prevent CVD remain uncertain. As such, our findings of an endothelial nitric oxide synthase (eNOS) redox switch mechanism as hypothesized above may provide a key biomarker to better predict the efficacy.

**Figure 1:**
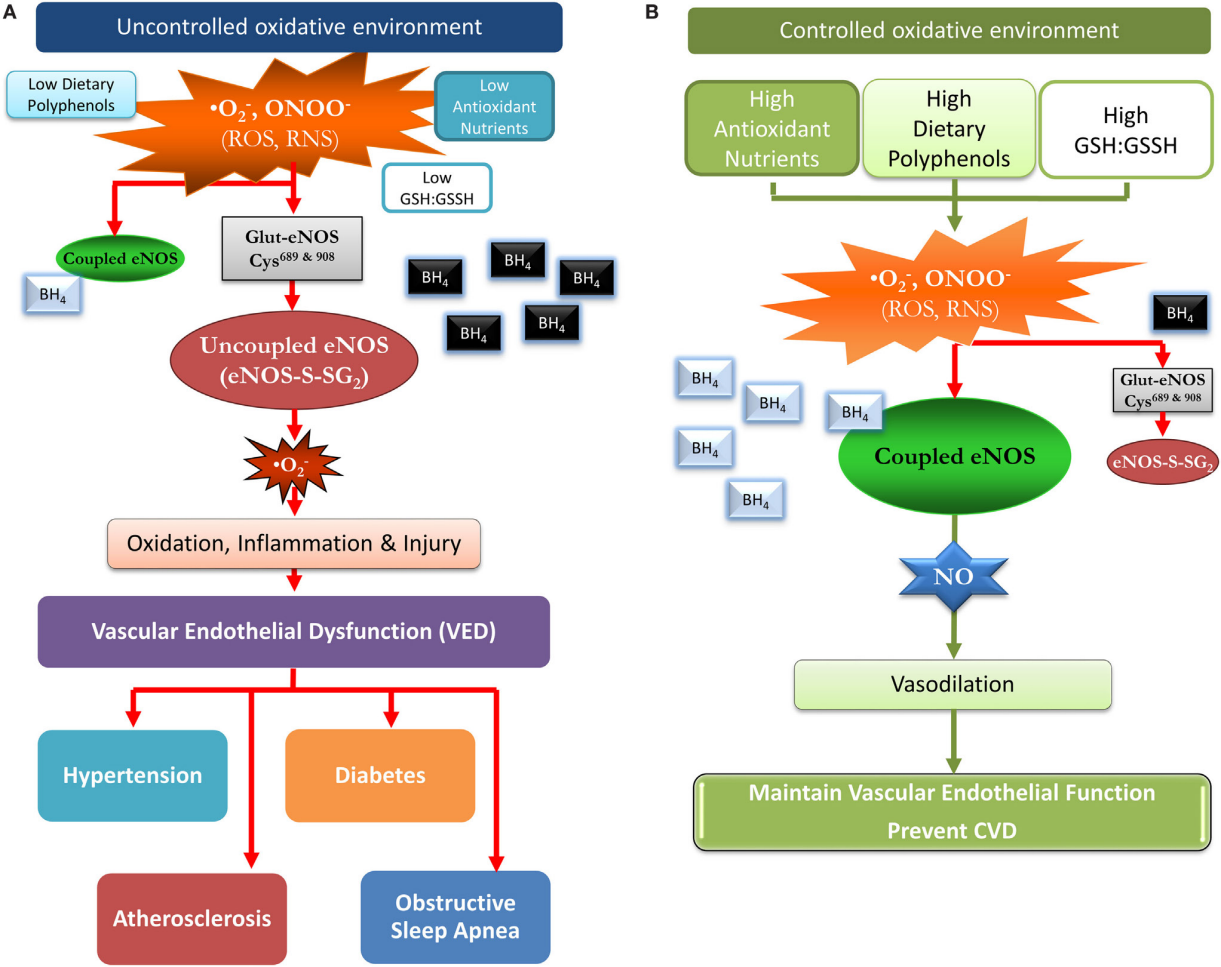
A proposed model of how a favorable redox environment prevents vascular endothelial dysfunction and possibly the risk for cardiovascular disease in an uncontrolled and controlled redox environment. **(A)** Uncontrolled oxidative environment: chronic low intakes of antioxidant nutrients and dietary polyphenols/flavonoids can result in a low glutathione (GSH):GSSH ratio and a diminished capacity to scavenge reactive oxygen species (ROS) and reactive nitrogen species (RNS). The oxidative burden favors *S*-glutathionylation (oxidation) of cysteine residues 689 and 908 in endothelial nitric oxide synthase (eNOS) (eNOS-*S*-SG_2_), and BH_4_ is oxidized (black with white font), leading to uncoupled eNOS, and superoxide $\left({\text{O}}_{2}^{\cdot -}\right)$ formation. Thus, vasodilation is impaired due to loss of eNOS function with a pivotal switch from production of the vasodilator nitric oxide (NO) to the vasoconstrictor superoxide. Over time, this may contribute to vascular disease, intestinal ischemia, coronary ischemia, hypertension, diabetes, and hyperlipidemia. **(B)** Controlled oxidative environment: dietary flavonoids/polyphenols and antioxidant nutrients promote a thiol rich (high GSH:GSSH ratio) pool serving to scavenge ROS and RNS, thus, preventing or reversing protein *S*-glutathionylation (cysteine oxidation). This maintains unoxidized BH_4_ (silver with black font) levels and eNOS coupling, producing NO which promotes vasodilation and reduces vascular endothelium mediated inflammation/injury. This more favorable environment may over time, delay the progression to vascular disease, and other chronic disease.

Therefore, our hypothesis is that: (1) the reversible oxidative process of protein *S*-glutathionylation (Pr-*S*-SG) of eNOS, that serves as a molecular switch modulating the vascular function through the balance of nitric oxide (NO) and superoxide production of eNOS (26), is critical in the onset of VED which is a central mechanism leading to CVD and (2) that this redox switch could be modulated by dietary factors and antioxidants reducing the risk of CVD onset. Thus, this review focuses on a novel mechanism of vascular redox regulation and the potential role of nutrition and antioxidant supplementation in modulating this process and preventing VED and subsequent CVD, see Figure 1.

## Oxidative Stress, eNOS Uncoupling, and VED

**Figure 2:**
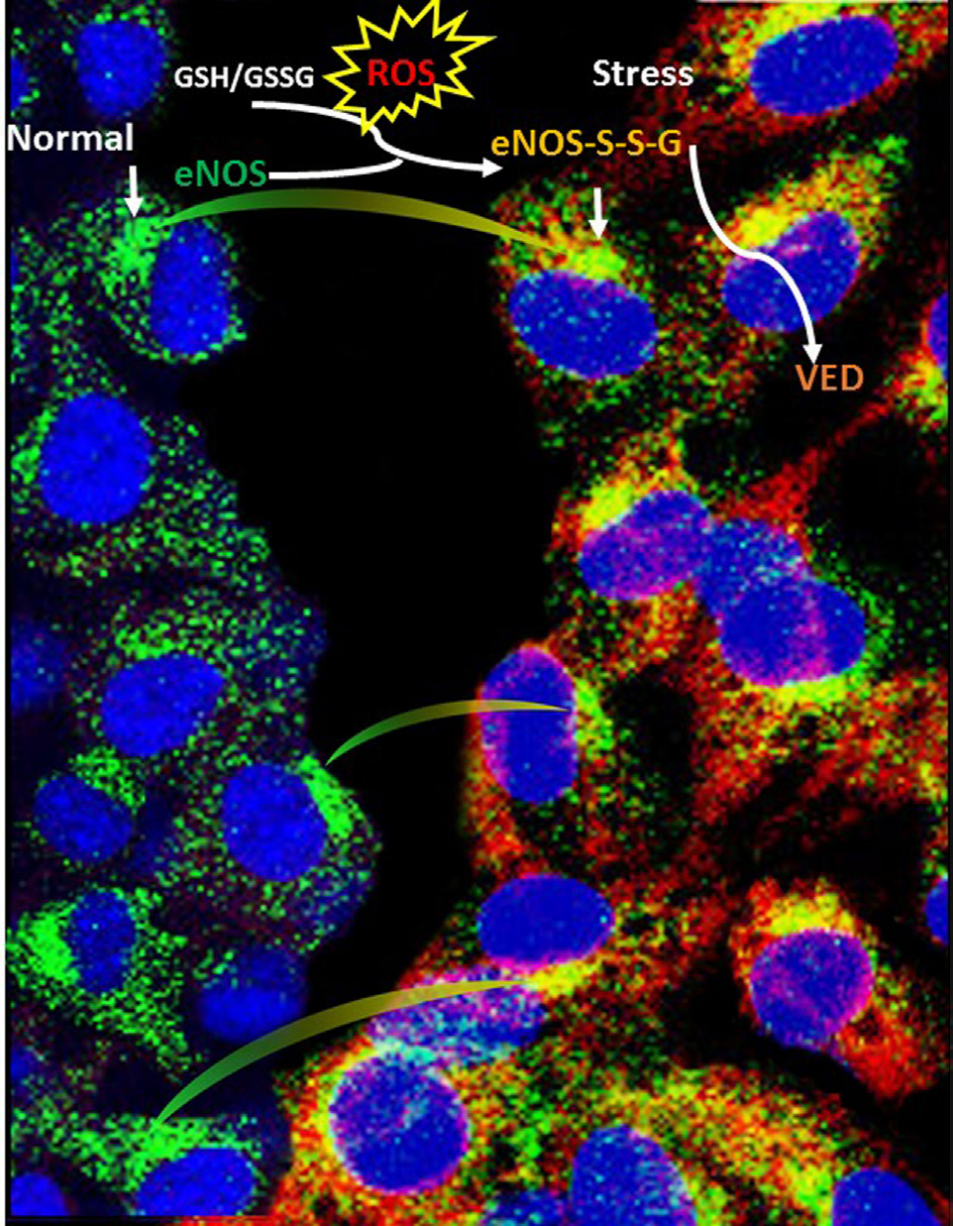
Schematic illustration depicting the mechanism of eNOS *S*-glutathionylation in endothelial cells. *S*-glutathionylation in endothelial cells (superimposed yellow fluorescence—eNOS-*S*-SG) due to endothelial nitric oxide synthase (eNOS) (green—down arrow) and glutathionylated eNOS (red—down arrow) when probed with general GSH antibody. A regulatory switch happens with physiological stress (oxidative stress), often during disease conditions, where eNOS makes superoxide in its glutathionylated form (blood vessel constriction), instead of nitric oxide (blood vessel dilation) compared with normal (healthy) conditions.

An imbalance between the physiological manifestations of reactive oxygen species (ROS) and the ability of the body to detoxify the free radicals leads to a state of oxidative stress, and has many effects on cellular processes. The normal redox state of cells is challenged due to the formation of free radicals and non-radical ROS including superoxide $\left(O_{2}^{\cdot -}\right)$ and hydroxyl radicals (^⋅^OH) as well as hydrogen peroxide (H_2_O_2_) (27). Oxidative stress is involved in the progression of heart and lung diseases (28, 29), cancer (30), Alzheimer’s, and neurodegenerative diseases in humans (21). Vascular oxidative stress occurs through a series of pathways including; eNOS *S*-glutathionylation (eNOS-*S*-SG; eNOS uncoupling), see Figure 2, xanthine oxidase, and nicotinamide adenine dinucleotide phosphate oxidase (27, 31). Conversely, although not discussed here, ROS can be beneficial as a signal transducer in phagocytosis, exercise, and prevention of aging (32), as well as many other conditions/states.

In male C57BL/6J mice, high fat diet induced obesity resulted in eNOS uncoupling in the perivascular adipose tissue (33). While this could suggest a dietary link to eNOS uncoupling in some tissues, it may also suggest a link with obesity and eNOS uncoupling; although a poor diet did cause the obesity. Pr-*S*-SG is a reversible cysteine modification due to oxidative stress. The GSH:GSSG ratio status and its involvement in converting sulfonic acids to *S*-glutathione mixed disulfides support the reversible Pr-*S*-SG mechanism and its subsequent regulation of several redox sensitive proteins (34, 35). Pr-*S*-SG is an emerging molecular marker for CVD due to its involvement in several of the physiological processes related to CVD (36, 37). Similarly, eNOS-*S*-SG can function as a molecular switch shifting eNOS production from NO to superoxide $\left(O_{2}^{\cdot -}\right)$ synthesis (26, 38). VED is primarily caused by decreased capacity of eNOS to produce NO (decreased bioavailability of NO) (29, 39). A novel reversible mechanism of eNOS uncoupling triggered by oxidative stress with the demonstration that *S*-glutathionylation of critical cysteine residues (residue 689 and 908) of eNOS triggers decreased NO production and increased $O_{2}^{\cdot -}$ production leading to VED has been reported (26). This work has more recently been translated to humans demonstrating eNOS *S*-glutathionylation and uncoupling in aortic tissues of patients undergoing cardiovascular surgery and heart transplantation (40, 41).

Another key mechanism of eNOS uncoupling is through oxidative depletion of its cofactor tetrahydrobiopterin (BH_4_); considered an antioxidant in itself (42). The redox sensitive cofactor BH_4_ is also essential for NO production from eNOS, and the BH_4_ levels are stabilized by vitamin C (ascorbate) (43, 44). Folate and cellular reducing equivalents are also required for its de novo synthesis and salvage pathways (44). Furthermore, it has been shown that eNOS *S*-glutathionylation can trigger BH_4_ depletion resulting in further eNOS uncoupling with loss of NO production and enhanced superoxide generation. BH_4_ depletion has been shown to occur in a variety of animal and human disease models (45, 46), although human studies of oral BH_4_ replacement therapy have yielded mixed results (47). Interestingly, BH_4_ supplementation (10 mg/kg/day) significantly decreased various types of congenital heart defects in the embryos of pregestational diabetic mice (42), suggesting the importance of sufficient antioxidant and anti-inflammatory compounds during pregnancy. There are currently no known food sources of BH_4_ although intestinal microbiota may be a source (48), suggesting future research may show low levels of BH_4_ producing bacteria in CVD patients or in other chronic conditions/diseases.

Over the last two decades, it has been demonstrated that there are two major pathways of NO production, one through endogenous vascular endothelium *via* l-arginine conversion by eNOS coupling, and the other through dietary intake of nitrate, nitrites, and antioxidants. At physiological concentrations, NO increases cyclic GMP levels in blood vessels, which inhibit contractile protein function to cause smooth muscle relaxation (49). Furthermore, endothelium-derived NO also controls the production of coagulation factors that regulate platelet activity, the clotting cascade, and the inflammatory process. NO exerts hypotensive, antiplatelet, and cytoprotective effects (50). The l-arginine pathway becomes dysfunctional with age, whereas the dietary pathway of NO formation from nitrate or nitrites does not appear to be affected by age-related factors. Thus, a diet rich in nitrate/nitrite along with antioxidants to facilitate reduction to NO, may be able to overcome or compensate for the insufficiency in endothelium-derived NO (23). There may be a need to revisit dietary nitrite and nitrate recommendations (51) given the low intake of antioxidants in the United States diet (25).

Since both BH_4_ depletion and eNOS *S*-glutathionylation are caused by oxidative stress, there may be a concurrence of these two mechanisms of eNOS uncoupling in the VED that accompanies CVD. While recent reports in cellular models suggest that these pathways converge, their relationship in human disease remains unknown (52, 53). Studies have already shown the significance of oxidative cysteine modifications in modulating vascular function and as noted above the inclusion of poor nutrition as a risk factor for CVD (54). Therefore, therapeutic interventions to reverse VED and the subsequent progression to CVD could include nutritional compounds, dietary supplementation, or modification and this could address the regulatory mechanisms of eNOS uncoupling or provide alternative sources of NO from dietary nitrite.

## Dietary Antioxidants in Vascular Preservation

While there is no specific mention of protein *S*-glutathionylation, antioxidants, or anti-inflammatory compounds in AHA/ACC guidelines, diets such as MED, DASH, AHA, and USDA are relatively high in antioxidants and anti-inflammatory nutrients and compounds (55). The AHA/ACC recommendations are based on specific clinical outcomes, such as the blood lipid lowering effect of these diets (22). If the benefits of such dietary patterns, or choices, are related to higher antioxidant and anti-inflammatory capacity, then the guidelines related to optimal timing and portion control (56) may suggest frequent intakes (each meal) of antioxidants and anti-inflammatory compounds from food are better than larger doses at random times, or possibly overall lower intake levels.

In their review, Pastore and Piemonte (37) indicated that *S*-glutathionylation may protect cysteine residues in proteins and therefore are meant to be a temporary event; preserving further oxidation of the proteins until cellular conditions return to normal. Therefore, chronic, or uncontrolled, *S*-glutathionylation may be more concerning. This process could then be similar to inflammation; acute (temporary) inflammation is a physiological response to stress or infection, but chronic inflammation is a pathophysiologic condition with negative health consequences (57). Cellular metabolism is designed to handle inherent ROS, in fact, the entire ROS and redox system, including Pr-*S*-SG, is an intracellular signaling system. Within this system, Pr-*S*-SG parallels phosphorylation making it a regulatory mechanism linking mitochondrial function (energy generation) to whole cell protein activity (58). This link offers some explanation as to why Pr-*S*-SG should be short term, to protect proteins during times of elevated ROS and nutrient antioxidants may also prevent the subsequent oxidation process.

The nutrient antioxidants include vitamin E, vitamin C, and selenium. The carotenoids (e.g., β-carotene) are major contributors but are not considered essential nutrients like the vitamins and minerals. Besides these dietary antioxidants, the most widely known plant antioxidants are other tocopherols (besides vitamin E: R,R,R-α-tocopherol), tocotrienols, carotenoids, and phenolic/polyphenolic compounds (phenolics) (55). Phenolics from fruits and vegetables are the most studied in terms of their antioxidant capacity and ability to modulate disease progression, see recent review (59). The most widely known in relation to CVD, are the green tea catechins [epicatechin, epicatechin-3-gallate, epigallocatechin, and epigallocatechin-3-gallate (EGCg)] (60), cocoa polyphenols (approximately 37% catechins, 4% anthocyanins, and 58% proanthocyanidins) (61), and resveratrol from grapes/wine (62, 63). For the most part, phenolics are water soluble, which may explain their low bioavailability and fast clearance from the blood (55). Because there is some evidence to suggest resveratrol can benefit cardiovascular health (64–66), more work is warranted to explain which metabolites have beneficial properties, or if some alternative mechanism (e.g., gut microflora) is involved. Pharmacokinetic data for antioxidant compounds (from foods and supplements) may add some evidence to the importance of meal timing, and frequency, in CVD prevention (56) by suggesting regular intakes of antioxidants may offer the greatest benefit to CVD prevention.

A Cochrane systematic review and meta-analysis have shown that green and black tea may be beneficial for the primary prevention of CVD, due to their lowering effects on LDL cholesterol and blood pressure (67), although longer term studies are required to make clearer recommendations. Cocoa products, e.g., chocolate, over the short term (approximately 4 months) have a blood pressure lowering effect (2–3 mmHg) (68), however, an earlier meta-analysis found that the effect may only be those who are hypertensive (69). An LDL cholesterol and total cholesterol lowering effect of cocoa products were also found in a separate meta-analysis (70). Interestingly, although the AHA/ACC dietary guidelines promote more fruits and vegetables, the Cochrane systematic review showed no clear benefit of fruits and vegetables on the primary prevention of CVD risk factors due to the inability to separate fruits and vegetables from other dietary and lifestyle components (71).

Green tea (6 g) has been shown to improve flow-mediated endothelium-dependent vasodilation (FMD) in healthy subjects within 30 min after consumption (72). In young relatively healthy Korean smokers, 8 g of green tea per day for 2 weeks resulted in significantly improved (7.2 ± 2.8 vs 9.3 ± 2.4, *p* < 0.001) FMD, and increased (78.6 ± 72.6 vs 156.1 ± 135.8/ml, *p* < 0.001) the number of endothelial progenitor cells (EPC) (73). In patients with chronic renal failure, 5 g of green tea/day for 1 month, significantly improved FMD but not EPC numbers (74). In hypertensive patients, 12 mg catechins (from 500 mg of black tea), twice daily for 8 days, counteracted the negative effects of a fatty bolus of whipping cream (1 g fat per kg body weight) on FMD, and increased circulating angiogenic cells, vs control, possibly *via* the activation of eNOS (75). Cocoa is less studied; however, blood pressure decreases and better NO bioavailability may be related to flavanols derived from cocoa, see review (76), as well as inhibition of platelet aggregation, see review (77). Cocoa may even have more beneficial effects in older subjects, compared with young (78), and improve brachial artery hyperemic blood flow after 6 weeks *via* a decrease in soluble vascular cell adhesion molecule-1, in postmenopausal hypercholesterolemic women (79). These studies show how rapidly green tea and cocoa have beneficial cardiovascular effects, and may indicate their use in secondary prevention; however, more studies are required in chronic conditions/diseases. Regardless, there is evidence that green tea consumption may reduce the risk of CVD and ischemic diseases, albeit dose dependent, but there was a higher risk of CVD in those who did not consume green tea (80).

The mechanisms of action for phenolics and cardiovascular benefits are well described in the literature. As an example, in rats with streptozotocin induced diabetes, green tea (5 g/kg body weight/day instead of water) was reported to increase BH_4_ levels and NO bioavailability, alleviating eNOS uncoupling, and reduced oxidative stress (81). NO itself may also be a potential signal for protein glutathionylation (82). Therefore, NO is necessary for vasodilation and cardiovascular health but under certain conditions becomes a pro-oxidant [see review (83)].

The decline in mortality related to CVD since 1950 may be due, in some small part, to folic acid and vitamin B_6_ fortification, after which the United States Food and Drug Administration made fortification of folic acid mandatory in 1998 (to reduce homocysteine) (84). While the homocysteine hypothesis is not without its critics (85), there is some evidence to show that folic acid supplementation can improve endothelial function (86), and may even slow the decline of endothelial function in those with familial hypercholesterolemia (87). Regardless, this does suggest that long-term supplementation of the diet with appropriate nutrients can lower the risk for CVD. Modifiable risk factors for CVD are poor diet, sedentary lifestyle, obesity, overconsumption of alcohol, and tobacco use (88). Changes in consumer preferences are indirectly increasing awareness of plant antioxidants as more “natural” and “non-GMO” sources are being sought to preserve foods and prolong shelf life (89). This may indirectly benefit CVD prevention, if the food chain is to begin producing foods with more phenolics, but may complicate future epidemiological studies. Phenolics may improve an unhealthy diet and may counteract some of the negative effects on blood flow and blood pressure. While there is no evidence to suggest phenolics can substitute for activity, prevention of eNOS coupling is among the benefits of exercise (90), as it increased iNOS and eNOS gene expression in endothelium (91). Some literature suggests green tea (and others) can reduce the effects of obesity (92); a risk factor for CVD. Excess alcohol beverage consumption may outweigh the benefits in relation to preventing CVD as many alcohol beverages contain phenolics (93). Finally, as described previously, green tea may counteract oxidant stress (prevention of the negative acute effects of smoking on blood flow) (73) and improve brachial artery flow-mediated dilation (a marker of endothelial function) in clinically stable patients with coronary artery disease (94).

Once considered food additives villain, nitrites and nitrates may be useful therapeutics providing alternative sources of NO under pathological conditions, see reviews of literature supporting this hypothesis (24, 95, 96). A more recent review also suggests nitrate/nitrate may improve endothelial function, blood pressure, and possibly alleviate metabolic syndrome (97). Another interesting area of research is in novel, or modified, antioxidants. These would be derivatives of naturally occurring phenolics to improve bioavailability and function (or other desired change), compounds not typically seen as antioxidants such as phospholipids and peptides, and esters of EGCg, eicosapentanoic acid (EPA), and docosahexanoic acid (DHA) (55). These esters of EPA and DHA seem to combine antioxidant and anti-inflammatory properties. More work in lipophilic antioxidant compounds such as lipoic acid (a cofactor for the pyruvate dehydrogenase complex), which has shown some promise as it may be effective at reducing redox involved vascular calcification is also warranted. The dual approach of hydrophilic and lipophilic antioxidants may produce better results due to the lipophilic compounds avoiding the liver after consumption (first pass effect).

The protective role of antioxidants, from fruit and vegetable intake, in chronic disease prevention seems logical, however, studies of supplements are varied and sometimes contradictory (98, 99), and as such the role of antioxidants in preventing atherosclerosis is still debated (98). It may be somewhat unfair to rule out the entire antioxidant hypothesis because isolated and concentrated forms of particular antioxidants did not work in prior clinical trials. There remain questions regarding what particular antioxidants are most potent, and what doses and ratios of antioxidants can be efficacious. The potential beneficial effects of as well as the multitude of dietary molecules, from each specific plant/fruit/vegetable which contribute to the overall antioxidant properties of the diet remain unknown (100). There is an effort to develop biomarkers to measure antioxidant status and oxidative stress (101), and this endeavor deserves more attention as it may help answer antioxidant-related questions. Another interesting approach is to enhance the production or delivery of BH_4_, a key molecule in maintaining eNOS coupling. Apart from *de novo* intracellular synthesis, certain intestinal microbiota may provide a unique exogenous source (48). This suggests there could be a gut microflora–antioxidant connection (from fruit and vegetable intake or individual antioxidant molecules); however, little is known about how the microbiome effects CVD and this field is still in its infancy (102). The microbiota link also could mean that the numerous dietary antioxidants, both nutrient and non-nutrient, may act *via* indirect mechanisms.

## Oral Microbiome, Oxidative Stress, and CVD

As described above, the microbiome may be of importance in providing specific antioxidants or in increasing their absorption. Recent findings in mice suggest that the gut microflora regulates GSH metabolism (103). The link between disease states and the microbiome is gaining importance, although more work is needed. An intriguing area of research is in the effect of diet and dietary components such as antioxidant content on the metabolome, as the foods consumed are the substrate for microbiome metabolism (104). While gut microflora have gained recent attention, the oral microbiome may also be important in CVD.

No direct links between oral health and CVD have been made but periodontal disease and CVD share risk factors (105). One intriguing area is how the oral microbiome may affect NO homeostasis. While the role of the gut microbiome in contributing to conversion of endogenous or ingested nitrate to nitrite has been well studied, a lesser known mechanism that has recently come into focus is the entero-salivary nitrate–nitrite-NO pathway. Approximately 25% of the circulating nitrates are sequestered by the salivary glands and are released into the oral cavity, resulting in concentrations 10-fold higher than plasma levels. Previously, the only beneficial role of salivary nitrates was believed to be reducing dental caries. Cariogenic organisms such as *Streptococcus mutans* and *Lactobacilli* are susceptible to nitrite and this chemical is both bactericidal as well as antiacidogenic (106). Beetroot juice has been demonstrated to have potent anticariogenic effects that are mediated through the entero-salivary circuit (107). However, emerging evidence indicates an antihypertensive role for oral commensals, one that is mediated through the entero-salivary nitrate–nitrite pathway. Certain oral commensals (notably, *Veillonella atypica, V. dispar, Actinomyces odontolyticus, A. naeslundii, A. viscosus, A. oris, Rothia mucilaginosa, R. dentocariosa, Staphylococcus epidermidis, Granulicatella adiacens, Haemophilus parainfluenzae, Neisseria flavescens, N. mucosa, N. sicca, N. subflava, Prevotella melaninogenica*, and *P. salivaei*) are known to reduce salivary nitrates to nitrite (108, 109). Thus, using an antibacterial mouthwash negates an increase in circulating nitrite in response to an oral nitrate load (110). Furthermore, volunteers who did not swallow their saliva for a 3-h period following a dose of nitrate did not experience a reduction in blood pressure as anticipated. This phenomenon has also been observed in the physiological state, in the absence of nitrate supplementation. Volunteers who used mouthwash on a daily basis demonstrated a 90% decrease in salivary nitrite levels, accompanied by a mean increase of 3.5 mmHg in blood pressure (111). Consequently, there is an emerging body of work that questions the indiscriminate and unregulated use of antibacterial mouthwash, especially among individuals with risk factors for CVD. However, modifying mouthwash by the addition of nitrite and possible reduction in antibacterial compounds, or even a nitrite-based mouthwash, may reduce dental caries and maintain a beneficial oral microbiome, and reduce CVD risk.

## Linking Inflammation and Oxidation

Inflammation is associated with oxidative stress (112). It is known for many years that systemic chronic inflammation (especially low grade) and CVD are linked, but especially when associated with obesity, see review (113). Chronic inflammation in the arterial wall leads to intra- and extra-vascular inflammation and ultimately results in vascular damage (114). While the original events that caused chronic inflammation could be any number of factors, including systemic inflammation can contribute to localized inflammation and CVD. Cohen et al. associated chronic stress (a risk factor for CVD) (115) with increased inflammation, *via* glucocorticoid receptor resistance, resulting in a negative impact on the immune system (116).

Inflammation is a concept which is recognized in the public domain and there is a multitude of online information related to anti-inflammatory diets and foods (112, 113). The most notable anti-inflammatory nutrients are the n-3 polyunsaturated fatty acids (n-3 PUFA). Convincing evidence suggests diets low in n-3 PUFA (low ratio of n-3 PUFA to other fats) are a cause of chronic inflammation and contribute over time to chronic disease. This topic has been greatly reviewed; for examples, see Ref. (57, 113, 117, 118). Furthermore, the overall macronutrient composition of a food/meal can determine its inflammatory properties. It is known that higher postprandial glucose levels may predict CVD (and diabetes) but these glucose spikes may also be inflammatory (and oxidative) (113, 117, 118); while the associated insulin response being anti-inflammatory (118). There is limited information on the anti-inflammatory actions of plant compounds in humans (113) and this represents a large knowledge/research gap in CVD prevention and treatment. However, Abdallaha and Esmat highlight the potential for using plants in the treatment of inflammation. They show that from the flowering aerial parts of *Zygophyllum simplex* L, a plant used in Arabic regions to treat inflammatory conditions, five major phenolic compounds were isolated. Each compound had somewhat different characteristics and exhibited *in vitro* anti-inflammatory properties at different concentrations; the most potent was at a concentration of 1 µM (119). It is of importance to understand the pharmokinetics of these, and other compounds, such as curcumin (120) and other commonly used herbs and spices (121), and investigate which compounds target localized or systemic inflammation in humans.

## Conclusion

**Figure 3:**
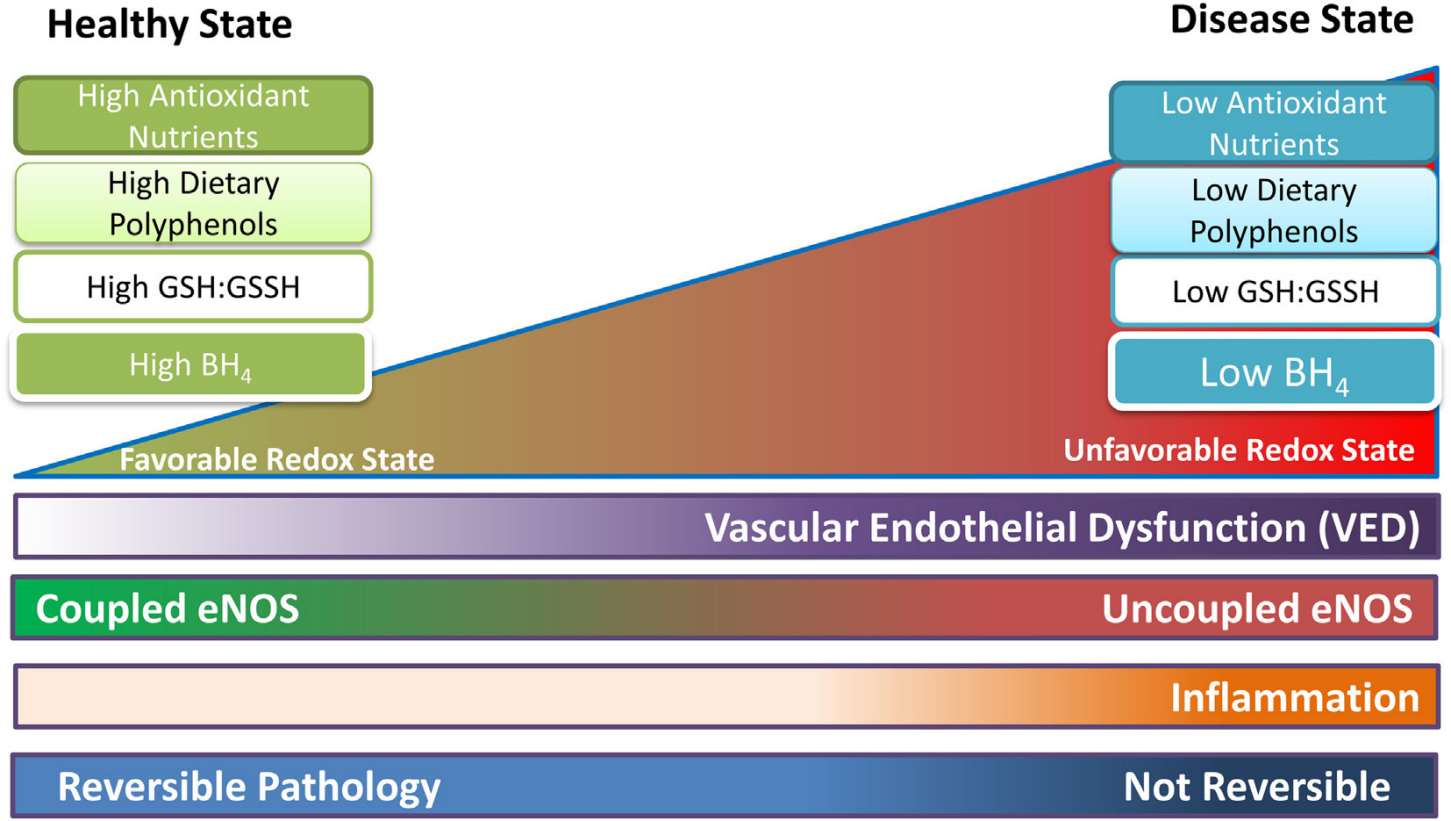
Proposed representation of progression of endothelial function as the redox state becomes less favorable. In the healthy state coupled endothelial nitric oxide synthase (eNOS) dominates as oxidative species and free radicals are handled by the high reductive capacity from dietary antioxidants. Tetrahydrobiopterin (BH_4_) is present in sufficient quantities. Uncoupled eNOS is present but is transient (high coupled eNOS:uncoupled eNOS ratio), and mostly occurs in response to localized fluctuations in the redox state. As the pathology shifts to the right, uncoupled eNOS is becoming more common (lower coupled eNOS:uncoupled eNOS ratio); the redox state is less able to cope with oxidative stress; and vascular endothelial dysfunction (VED) begins. BH_4_ levels are reduced leading to further oxidative damage and possibly inflammation. Once progression of the disease increases (shifts to the right of the figure), uncoupled eNOS is permanent (low coupled eNOS:uncoupled eNOS ratio), BH_4_ levels are at their lowest, and coupled eNOS is at low levels. Now oxidative reactions dominate and VED is established, leading to CVD and other chronic conditions/diseases. Up to a certain point, before VED is established and the pathology has resulted in permanent changes, the process may be reversible with lifestyle changes and medication.

In all probability, no one antioxidant or anti-inflammatory compound from food or supplements may provide the key to balancing the redox state and preventing or managing VED and CVD. A diet containing an abundance of antioxidants and supplements (ascorbate, green tea or cocoa polyphenols, other fruits and vegetables, etc.) may provide ability to limit oxidation, maintain a beneficial GSH:GSSH ratio, and other redox markers, preserving eNOS function and NO generation while preventing eNOS uncoupling and secondary superoxide production. The benefit of a diet higher in a multitude of antioxidant compounds is that a favorable redox environment is maintained *via* multiple pathways (mechanisms of action). It is ironic that although current dietary guidelines indirectly promote a diet higher in antioxidants, the major limitation in recommending antioxidant-based dietary guidelines is the inadequate evidence to support the same. Therefore, well-designed human studies are required to understand the mechanism of action of such diets or particular antioxidants. These studies would then scientifically justify the inclusion of certain foods or supplements in dietary guidelines to preserve vascular function, prevent eNOS uncoupling, and maintain a beneficial redox state, with the end goal of preventing or ameliorating the development of CVD, see Figure 3.

## Author Contributions

SV initiated the process upon receiving an invitation from the respective journal, SV acquired data by performing molecular/cellular experiments, SV and OK conceived the idea for the manuscript, acquired, and interpreted the relevant literature, created the first draft, revised subsequent drafts, provided final approval, and are accountable for all aspects of the work. JZ and SV provided insights of eNOS function, contributed to the editing and manuscript revision. SV, JZ, RK, PK, and NA provided insights on the mechanistic role and heart health implications of dietary antioxidants and anti-inflammatory compounds, provided final approval and are accountable for all aspects of the work.

## Conflict of Interest Statement

The authors declare that the research was conducted in the absence of any commercial or financial relationships that could be construed as a potential conflict of interest.
